# Safety and Immunogenicity of 1 or 2 Additional Doses of the Adjuvanted Recombinant Zoster Vaccine Administered 5–6 Years After Primary Vaccination in Adults ≥50 Years

**DOI:** 10.1093/ofid/ofag282

**Published:** 2026-05-04

**Authors:** Rafael Leon, Javier Diez-Domingo, Charles Andrews, Clovis Arns da Cunha, Eun-Ju Choo, David Shu Cheong Hui, Anthony L Cunningham, Takashi Eto, Giancarlo Icardi, Shelly A McNeil, Airi Põder, Pavel Kosina, Lars Rombo, Tino F Schwarz, Juan Carlos Tinoco, Chong-Jen Yu, Jing Wang, Jyoti Soni, Manyee Tsang, Ana Strezova, Bruno Salaun, Agnes Mwakingwe-Omari, the Zoster-049 Study Group, Abul Kashem Munir, Agnes Csuth, Agnes Himpel-Boenninghoff, Aino Forsten, Airi Poder, Ake Olsson, Alain Baty, Alain Boye, Alen Jambrecina, Alex Rodríguez Badia, Alexander Thompson, Andrea Gori, Anitta Ahonen, Anna Vilella Morato, Anthony Cunningham, Antje Dahmen, Axel Schaefer, Azhar Toma, Barry Lubin, Beate Moeckesch, Beatrice Gerlach, Benita Ukkonen, Benjamin Lasko, Benoit Daguzan, Bernhard Schmitt, Bo Liu, Brian Webster, Bruce Rankin, Calvin Powell, Carlos Brotons Cuixart, Carol Pretswell, Catherine Vaillancourt, Cecil Farrington, Charles Andrews, Chester Fisher, Chigomezgo Munthali, Chiu-Shong Liu, Chong-Jen Yu, Christian Duroy, Christian Schubert, Christiane Klein, Christine Cerna, Christine Grigat, Christophe Genies, Christopher Lucasti, Cláudia Murta de Oliveira, Claus Keller, Clovis Cunha, Concepcion Nunez Lopez, Covadonga Caso, Cristiano Zerbini, Dae Won Park, Damien Mcnally, Dan Curiac, David Francyk, David García Vidal, David Shu Cheong Hui, Denis Taminau, Domenico Montu, Dominique Saillard, Donald Quinn, Duane Wombolt, Edmund Kwok Yiu Sha, Elina Sirnela-Rif, Elisabeth Barberan, Eric St-Amour, Eriko Kinugasa, Ernie Riffer, Essam Abdulhakim, Eugene Athan, Eun-Ju Choo, Eva Ackefelt Frick, Felix Proepper, Ferdinandus de Looze, Francesco Schioppa, François Brault, Frederick Martin, Gabriele Illies, Georg Plassmann, George Freeman, George Raad, Gerald Shockey, Giancarlo Icardi, Giuseppe Fiore, Guglielmo Migliorino, Guy Tellier, Hanna Karhusaari, Hans-Joachim Koenig, Hee Jin Cheong, Hiroaki Ogata, Hirohiko Sueki, Holly Dushkin, Hsiao-Ting Chang, Huey-Shinn Cheng, Hyo Youl Kim, Ignacio Bardón Fernández-Pacheco, Ilkka Seppa, Irina Zahharova, Iris Gorfinkel, Isaac Marcadis, Isabelle Schenkenberger, Jacob Lee, Jan Dutz, Janice Patrick, Javier Diez-Domingo, Jean Beytout, Jean-Sebastien Gauthier, Jeannette Janzen, Jeffrey Zacher, Jérôme Nuel, Jessica Claveau, Jin-Soo Lee, Joachim Minnich, Joachim Sauter, Joakim Aronsson, Joan Rothenberg, Johan Sanmartin Berglund, John Earl, John Ervin, Jonathan Staub, Jonathan Wilson, José Luiz Neto, José Ramón Méndez Rivas, Jose-Fernando Barba-Gómez, Josef Grosskopf, Juan Carlos Tinoco, Juergen Berger-Roscher, Juergen Schmidt, Juergen Stockhausen, Juergen Wachter, Jukka Markkula, Junya Irimajiri, Jurij Eremenko, Kae Kobayashi, Karl Wilhelm, Karlis Pauksens, Katarina Berndtsson Blom, Ken Heaton, Kenjiro Nakamura, Kyong Ran Peck, Lars Rombo, Lauri Peltonen, Laurie Breger, Lluis Martínez Via, Loïc Boucher, Luciano Goldani, M Luisa Rodrguez de la Pinta, Mahadev Ramjee, Maija Rossi, Manuel Terns Riera, Marc Dionne, Margaret Rhee, Maria Giuseppina Desole, Maria Hemming-Harlo, Maria Maestre Naranjo, Marita Paassilta, Marjaana Sipila, Mark Turner, Marshall Freedman, Marta Aldea Novo, Martin Lundvall, Martin Van Cleeff, Mary Beth Manning, Matthew Finneran, Maximilian Kropp, Megumi Inoue, Meral Esen, Merce Perez Vera, Michael Adams, Michael Mueller, Michael Redmond, Miia Virta, Monika Hamann, Murdo Ferguson, Nell Wyatt, Nicolas Galerne, Nicole Toursarkissian, Niklas Bengtsson, Noah Vale, Olli Henriksson, Otso Arponen, Outi Laajalahti, Pascal Hanrion, Patrice Nault, Patrick Robert, Paul Hartley, Paul Ivan, Paula Gyllemark, Pauliina Paavola, Pavel Kosina, Pavel Naplava, Pekka Koskinen, Pembe Ozunlu, Peter Eizenberg, Peter Gal, Peter Levins, Petr Dite, Philippe Remaud, Piero Barbanti, Pierre André Ferrand, Pierre Lachance, Pierre-Alain Houle, Pyrene Martinez Piera, Ralf Freese, Rie Kuroki, Robert Lipetz, Robert Rosen, Roman Chlibek, Samir Purnell-Mullick, Satu Kokko, Scott Polster, Shari Rozen, Shelly McNeil, Shin Suzuki, Shinn-Jang Hwang, Silvia Narejos Perez, Spyridon Miyakis, Srikanth Malempati, Stephan Morscher, Stephanie Powell, Steve Mueller, Steven Geller, Suganthi Luci Magimaiseelan, Susan Datta, Susanna Koski, Susannah Eyre, Susanne Hoeltz-Roehrig, Suvi-Tuuli Simojoki, Sylvia Shoffner, Takashi Eto, Tamara Eckermann, Tark Kim, Terry Poling, Tetsuhiko Nagao, Thomas Horacek, Thomas Jung, Thomas Weinke, Tiina Haapaniemi, Tiina Karppa, Tiina Korhonen, Tino Schwarz, Tommaso Staniscia, Trevor Gooding, Uwe Kleinecke, Wayne Ghesquiere, Wilfred Yeo, William Ellison, Wilson Jacob, Xavier Farres Fabre, Yieng Huong, Young Goo Song, Yuji Naritomi, Agnes Mwakingwe-Omari, Alemnew F Dagnew, Amy Tan, Ana Strezova, Anne Schuind, Andrew Hastie, Bruno Salaun, Céline Boutry, Emeline de Viron, Emmanuel Di Paolo, Hao Wang, Huizi Zhang, Joon Hyung Kim, Kamal Al Shawafi, Lidia Oostvogels, Mamadou Drame, Martine Douha, Mélanie Gilbert, Meng Shi, Mohamed Amakrane, Mohd Tariq, Nurhan Albayrak, Olivier Godeaux, Paola Pirrotta, Toufik Zahaf

**Affiliations:** GSK, Wavre, Belgium; FISABIO, Valencia, Spain; Catholic University of Valencia, Valencia, Spain; IMA Clinical Research San Antonio, San Antonio, Texas, USA; Centro Medico Sao Francisco, Curitiba, Brazil; Soonchunhyang University Bucheon Hospital, Bucheon, South Korea; The Chinese University of Hong Kong, Hong Kong, China; Centre for Virus Research, Westmead Institute for Medical Research, Sydney, Australia; University of Sydney Institute for Infectious Diseases, Faculty of Medicine and Health, University of Sydney, Sydney, Australia; Hakata Clinic, Fukuoka, Japan; Hygiene Unit, IRCCS San Martino Policlinico Hospital, Genoa, Italy; Department of Health Sciences (DISSAL), University of Genoa, Genoa, Italy; Canadian Center for Vaccinology, IWK Health, NS Health, Halifax, Nova Scotia, Canada; Dalhousie University, Halifax, Nova Scotia, Canada; Tartu University Hospital, Tartu, Estonia; Department of Infectious Diseases, Charles University Faculty of Medicine in Hradec Kralove and University Hospital Hradec Kralove, Hradec Kralove, Czech Republic; Centre for Clinical Research Sormland, Uppsala University, Uppsala, Sweden; Institute of Laboratory Medicine and Vaccination Centre, Klinikum Würzburg Mitte, Campus Juliusspital, Würzburg, Germany; Hospital General de Durango, Durango, Mexico; National Taiwan University College of Medicine and National Taiwan University Hospital, Taipei, Taiwan; GSK, Rockville, Maryland, USA; GSK, Wavre, Belgium; GSK, London, UK; GSK, Wavre, Belgium; GSK, Rixensart, Belgium; GSK, Rockville, Maryland, USA

**Keywords:** additional doses, herpes zoster, immunogenicity, recombinant zoster vaccine, safety

## Abstract

**Background:**

A phase 3b extension of the ZOE-50/70 trials evaluated long-term efficacy, immunogenicity, and safety of the recombinant zoster vaccine (RZV) in participants ≥50 years, with a 6-year follow-up after completion of the primary studies. A subset of participants was evaluated for immunogenicity and safety of 1 or 2 additional doses administered 5–6 years after primary vaccination.

**Methods:**

Participants were randomized to 1 additional dose (1-additional dose group, n = 61), 2 additional doses (revaccination group, n = 60), or no additional vaccination (control, n = 119). Humoral and cell-mediated immunity were evaluated by antiglycoprotein E (gE) antibodies, gE-specific CD4[2+] T-cells, and memory B-cells. Reactogenicity was evaluated for 7 days postvaccination and overall safety was evaluated throughout the study. NCT02723773.

**Results:**

Anti-gE geometric mean concentrations (GMCs) were 10 000–11 500 mIU/mL in all groups preadditional vaccination. Geometric mean concentrations peaked at 1 month after 1 dose (73 834.4 and 79 419.8 mIU/mL in the 1-additional dose and revaccination groups, respectively), declined at Year 1, but remained above preadditional vaccination levels thereafter. Geometric mean concentration was 64 603.0 mIU/mL 1 month after the second dose in the revaccination group. Geometric mean concentrations in the control group were 8825.4 mIU/mL at Year 1 and 6858.8 mIU/mL at Year 6. The frequency of gE-specific CD4[2+] T-cells and memory B-cells followed a similar pattern. Pain and fatigue were the most common solicited adverse events. No serious adverse events related to RZV were reported.

**Conclusions:**

A single additional RZV dose elicited strong and durable humoral and cell-mediated anamnestic responses, with a reactogenicity and safety profile as established in primary studies.

**Clinical Trial Registration:**

NCT02723773.

## Teaser

A single additional dose of recombinant zoster vaccine elicited strong humoral and cell-mediated anamnestic responses, with a benefit/risk profile in line with that established in pivotal studies.

## Graphical Abstract

**Graphical Abstract:**
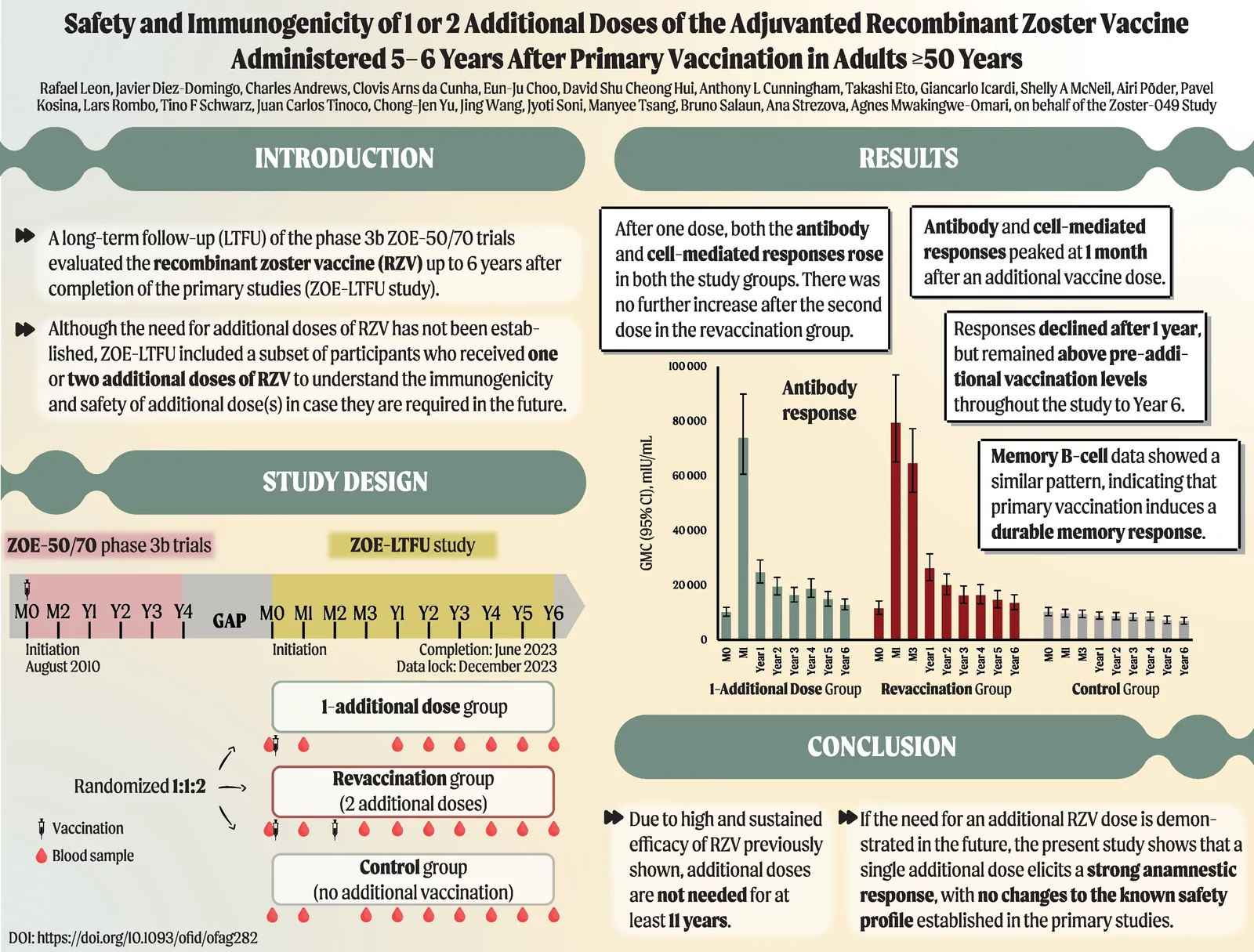

Herpes zoster (HZ) is caused by the varicella zoster virus (VZV) and occurs mainly in adults ≥50 years of age and in immunocompromised individuals [1, 2]. As the incidence of both HZ and associated complications rises with older age [3, 4], and the population is continuing to grow older in most parts of the world [5], many people are at risk for HZ and HZ-related complications.

The adjuvanted recombinant zoster vaccine (RZV; *Shingrix*, GSK) is composed of a recombinant VZV glycoprotein E and the *AS01_B_* Adjuvant System. It is approved in more than 70 countries for the prevention of HZ in adults ≥50 years and in adults ≥18 years at increased risk of HZ [6]. High vaccine efficacy (VE) of RZV was shown over 3–4 years of follow-up in 2 pivotal phase 3 trials, ZOE-50, conducted in adults ≥50 years of age, and ZOE-70, conducted in adults ≥70 years of age [7, 8].

A long-term follow-up (LTFU) extension study of the 2 pivotal studies (ZOE-LTFU) has been conducted in which participants who received RZV in ZOE-50 and ZOE-70 were followed up to approximately 11 years after primary vaccination [9]. We have previously reported long-term VE, safety, and immunogenicity in participants who received no additional vaccination in ZOE-LTFU, demonstrating that the vaccine offers durable protection against HZ and associated complications, with minimal waning of VE [9].

Although the need for additional doses of RZV has not been established, ZOE-LTFU also included individuals who received 1 or 2 additional doses of RZV in order to understand the immunogenicity and safety of additional dose(s) in case they are required in the future. In this article, we report the immunogenicity and safety of 1 additional dose (1-additional dose group) or 2 additional doses (revaccination group) of RZV administered 5–6 years after primary vaccination in ZOE-50/70.

## METHODS

### Study Design and Vaccine

The design of ZOE-LTFU (NCT02723773) has been described previously [9]. Briefly, ZOE-LTFU was a phase 3b extension study of the pivotal RZV phase 3 trials, ZOE-50 in participants ≥50 years (NCT01165177) and ZOE-70 in participants ≥70 years (NCT01165229). The protocol was approved by required independent ethics committees or institutional review boards and participants provided written, informed consent. The study was conducted in accordance with the Declaration of Helsinki and the principles of Good Clinical Practice. The study was sponsored by GSK.

**Figure 1.**
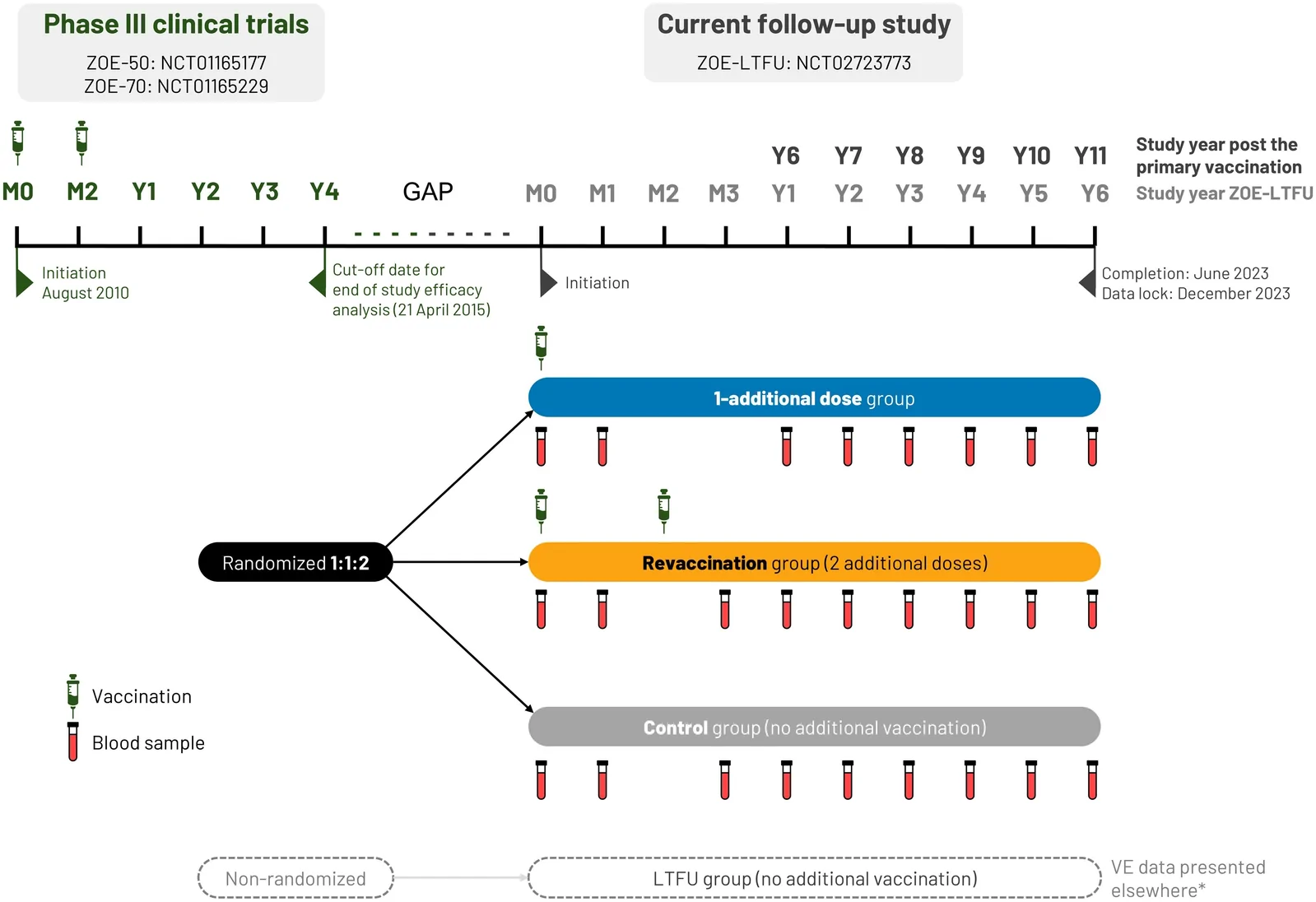
Study design. The gap period between the cutoff date for the end of study efficacy analysis in the ZOE-50/70 studies (21 April 2015) and the first visit in the ZOE-LTFU study varied between participants depending on the timing of their first visit in ZOE-LTFU. *Strezova et al [9]. Abbreviations: LTFU, long-term follow-up; M, month; VE, vaccine efficacy; Y, year.

Participants were eligible for the ZOE-LTFU study if they had received at least 1 dose of RZV in ZOE-50 or ZOE-70. Full eligibility criteria have been published previously [9]. The study included 4 participant groups (Figure 1). Most participants were included in the LTFU group; these participants had received either 1 or 2 doses of RZV in ZOE-50 or ZOE-70 and received no additional vaccination in ZOE-LTFU. Participants in the LTFU group were evaluated for efficacy as previously reported [9]; no data from this group are shown in the present publication.

The present publication focuses on immunogenicity and safety data in the other 3 study groups, which were randomized in a 1:1:2 ratio to receive either 1 additional RZV dose (1-additional dose group), 2 additional doses (revaccination group), or no additional vaccination (control group) in ZOE-LTFU (Figure 1). Participants were eligible for the randomized groups if they had received 2 doses of RZV in ZOE-50/70 and had not developed confirmed or suspected HZ during ZOE-50 or ZOE-70 or during the gap between the end of ZOE-50/70 and the start of ZOE-LTFU; additional criteria are shown in the Supplement. Participants in the randomized groups were enrolled from 3 preselected study countries that had readily available facilities for processing peripheral blood mononuclear cells for the cell-mediated immunity (CMI) analyses. The first participants who enrolled in these countries and met eligibility criteria for the 1-additional dose, revaccination, or control groups were included in 1 of the 3 groups.

Vaccine supplies were randomized within blocks by GSK using the MATerial EXcellence (MatEx) program. Participants were allocated to a study group by investigators using a randomization system on internet (SBIR), with a minimization procedure accounting for age at first vaccination in the ZOE-50/70 study (50–59, 60–69, and ≥70 years) and country. Minimization factors had equal weight in the algorithm. The study was open-label, although the laboratory team was blinded to treatment allocation.

RZV comprises 50 μg of recombinant VZV glycoprotein E (gE) and the *AS01_B_* Adjuvant System, which is a liposome-based adjuvant comprising 50 μg 3-*O*-desacyl-4′-monophosphoryl lipid A and 50 μg QS-21, a saponin from the *Quillaja saponaria* Molina tree [10].

### Outcomes

Outcomes related to long-term persistence of efficacy, immunogenicity, and safety have been reported previously [9]. This article focuses on the randomized groups (1-additional dose, revaccination, and control groups), reporting the secondary and tertiary objectives of immunogenicity, reactogenicity, and safety of the additional vaccine doses.

#### Assessment of Immunogenicity

Blood samples for immunogenicity assessment were taken on the first day of the study in all study groups (preadditional vaccination for the 1-additional dose and revaccination groups), at Month 1 in all study groups, Month 3 in the revaccination and control groups, and from Year 1 to Year 6 in all study groups (Figure 1). Humoral immunity (HI) was evaluated by the concentration of anti-gE antibodies measured by enzyme-linked immunosorbent assay, with a cutoff value of 97 mIU/mL. Cell-mediated immunity was evaluated in terms of the frequency of gE-specific CD4[2+] T-cells (CD4+ T-cells expressing at least 2 of 4 activation markers: tumor necrosis factor-α, interleukin-2, interferon-γ, and CD40 ligand) and the frequency of gE-specific memory B-cells. The frequency of gE-specific CD4[2+] T-cells was measured by intracellular cytokine staining and flow cytometry after stimulation with a pool of overlapping peptides covering the entire gE ectodomain sequence. gE-specific memory B-cells were quantified by flow cytometry using fluorescently labeled VZV gE protein as bait. Additional details are shown in the Supplementary Appendix (Methods and Supplementary Figure 1). Memory B-cells were characterized as live CD14^neg^/IgD^neg^/CD19^pos^/CD20^pos^/CD38^low^ cells.

#### Assessment of Reactogenicity and Safety

Participants recorded solicited injection site and systemic events for 7 days postvaccination in a diary. Spontaneously reported (unsolicited) adverse events (AEs), serious adverse events (SAEs), and potential immune-mediated diseases (pIMDs) were recorded for 30 days postvaccination. Medically attended AEs were recorded for 6 months postvaccination. Serious adverse events and pIMDs were additionally recorded up to 12 months postvaccination. Long-term safety throughout the study was evaluated in terms of (1) SAEs related to RZV, study participation (ie, study-mandated procedures and invasive tests), or to a concurrent GSK medication or GSK vaccine and (2) AEs and SAEs leading to withdrawal and related to RZV, study participation, a concurrent GSK medication or vaccine, or HZ-associated complications. Adverse events were defined as an untoward medical occurrence in a study participant, temporally associated with the vaccine, whether or not considered related to the vaccine; SAEs were defined as resulting in death, life-threatening, requiring or prolonging hospitalization, resulting in disability or incapacity or a congenital abnormality in the offspring of the vaccine recipient; pIMDs were a subset of AEs that include autoimmune diseases and other inflammatory or neurologic disorders of interest which may or may not have an autoimmune etiology.

### Statistical Analysis

Statistical analyses were performed using the Statistical Analysis Systems (SAS) Life Science Analytics Framework (SAS Institute Inc., Cary, NC, USA). The sample size of the overall study was driven by the VE analysis, which has been reported previously [9]. The present analysis of additional RZV doses was descriptive and did not require a sample size calculation. It was planned to enroll approximately 60 participants in the 1-additional dose and revaccination groups and approximately 120 participants in the control group.

The total vaccinated cohort (TVC) for the present analysis included all participants with valid data who were enrolled in the ZOE-LTFU and randomized to the 1-additional dose, revaccination, or control groups. Both the HI and CMI responses were assessed in the according to protocol (ATP) cohort for immunogenicity adapted for each yearly timeframe. The ATP cohort was defined for each time point and comprised participants who complied with protocol requirements in ZOE-50/70 and ZOE-LTFU and had immunogenicity data available for the time point considered. Reactogenicity and safety were assessed in the 1-additional dose and revaccination groups in the TVC.

The HI response was described in terms of the seropositivity rate (percentage of participants with anti-gE antibody concentration ≥97 mIU/mL [cutoff value]), geometric mean concentration (GMC) of anti-gE antibody, vaccine response rate (VRR; percentage of participants with postvaccination anti-gE antibody concentration ≥4-fold the cutoff value for initially seronegative participants and ≥4-fold the prevaccination value for initially seropositive participants), and mean geometric increase (MGI; geometric mean of within participant ratio of the anti-gE antibody concentration postvaccination/prevaccination). Prevaccination values for the calculation of VRR and MGI were from before primary vaccination in ZOE-50/70. Geometric mean concentrations were calculated by taking the anti-log of the mean of the log concentration transformations. Concentrations below the assay cutoff level were given an arbitrary value of half the cutoff level.

The frequency per million CD4 T-cells of gE-specific CD4[2+] T-cells was calculated as the difference between the frequency of CD4[2+] T-cells stimulated with gE peptides and those stimulated with culture medium alone. gE-specific memory B-cell frequencies were computed as frequencies per million total memory B-cells. Descriptive statistics (median, range, interquartile range) were reported for the frequencies of gE-specific CD4[2+] T-cells and memory B-cells.

## RESULTS

The ZOE-50/70 studies were conducted between August 2010 and July 2015; ZOE-LTFU was conducted between April 2016 and June 2023 (Figure 1). Data lock for ZOE-LTFU was December 2023.

### Participant Disposition and Demographics

**Figure 2.**
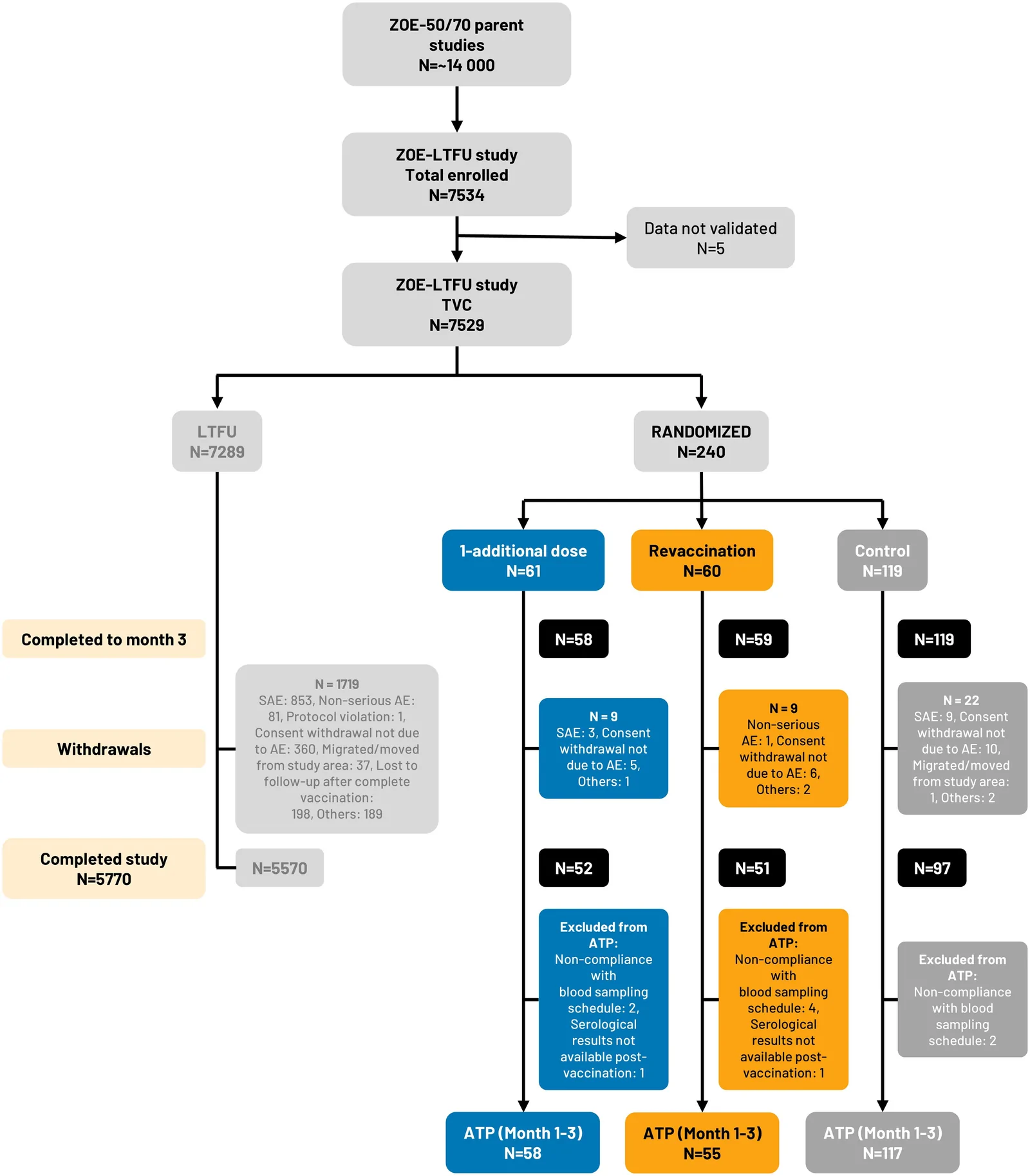
Participant disposition. Abbreviations: AE, adverse event; ATP, according to protocol; LTFU, long-term follow-up; N, number of participants; SAE, serious adverse event; TVC, total vaccinated cohort.

More than 14 000 participants from ZOE-50/70 were eligible for the ZOE-LTFU study. A total of 7529 participants were included in the TVC, of whom 61 were included in the 1-additional dose group, 60 in the revaccination group (of whom 55 received both additional vaccine doses), and 119 in the control group (Figure 2). The median interval between the second vaccine dose in ZOE-50/70 and the first dose in ZOE-LTFU in the 1-additional dose and revaccinated groups or the Month 0 visit in the control group was 5.2 (range 4.7–5.8), 5.3 (range 4.6–5.8), and 5.2 (range 4.6–5.6) years, respectively, in the TVC.

In the 1-additional dose, revaccination, and control groups, the mean age of participants at first vaccination in ZOE-50/70 was 65.5, 65.5, and 65.4 years, respectively, in the TVC (Table 1). A total of 49.2%, 60.0%, and 58.0%, respectively, were female, and almost all (>98%) were of White Caucasian or European heritage (Table 1).

**Table 1.** Summary of Demographic Characteristics in the TVC

Characteristic	1-Additional Dose N = 61	Revaccination N = 60	Control N = 119
Age at first vaccination in ZOE-50/70 studies, years, mean (SD)	65.5 (9.4)	65.5 (9.6)	65.4 (8.9)
Age at Month 0 in ZOE-LTFU study, years, mean (SD)	70.4 (9.2)	70.2 (9.4)	70.2 (8.6)
Gender, n (%)			
Female	30 (49.2)	36 (60.0)	69 (58.0)
Male	31 (50.8)	24 (40.0)	50 (42.0)
Ethnicity, n (%)			
American Hispanic or Latino	0	1 (1.7)	0
Not American Hispanic or Latino	61 (100.0)	59 (98.3)	119 (100.0)
Geographic ancestry, n (%)			
White (Caucasian or European heritage)	61 (100.0)	59 (98.3)	117 (98.3)
Other	0	1 (1.7)	2 (1.7)

Abbreviations: N, number of participants in each group; n, number of participants with stated characteristic; SD, standard deviation; TVC, total vaccinated cohort.

### Humoral and Cell-Mediated Immune Response

**Figure 3.**
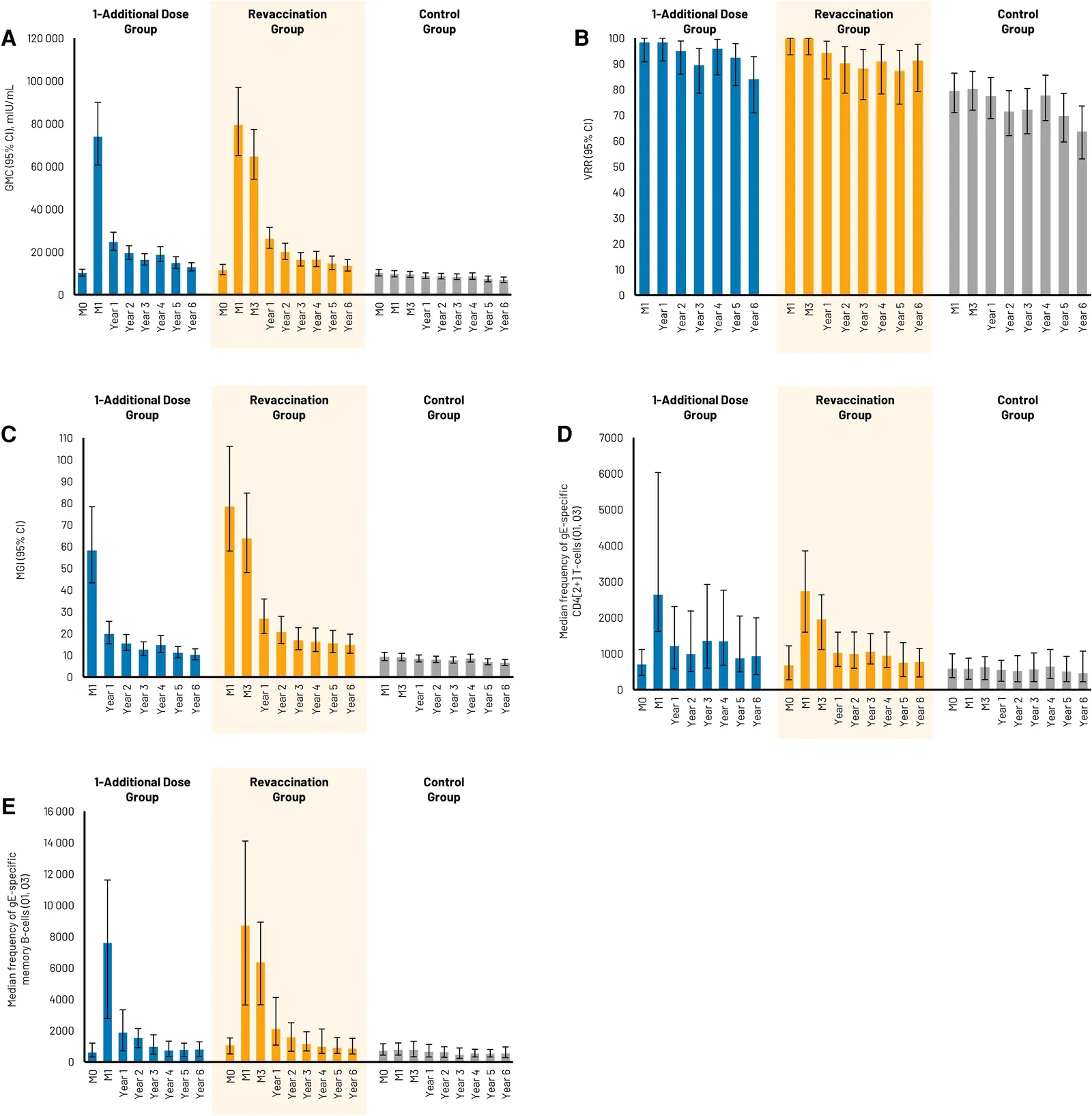
Immunogenicity assessments following additional RZV doses (adapted ATP cohort for immunogenicity). *A*, Shows the geometric mean concentration of anti-gE antibody. *B*, Shows the VRR for anti-gE antibody (VRR is the percentage of participants with postvaccination anti-gE antibody concentration ≥4-fold the cutoff value for initially seronegative participants and ≥4-fold the prevaccination value for initially seropositive participants; prevaccination values are from ZOE-50/70). *C*, Shows the MGI of anti-gE antibody (MGI is the ratio of the GMC postvaccination/prevaccination; prevaccination values are from ZOE-50/70). *D*, Shows the frequency of gE-specific CD4[2+] T-cells per million CD4 T-cells. *E*, Shows the frequency of gE-specific memory B-cells per million memory B-cells. Abbreviations: ATP, according to protocol; CI, confidence interval; GMC, geometric mean concentration; M0, month 0 (before additional vaccination in the 1-additional dose and revaccination groups); M1, month 1 (1 m after the first additional vaccine dose in the 1-additional dose and revaccination groups); M3, month 3 (1 m after the second additional vaccine dose in the revaccination group); MGI, mean geometric increase; N, number of participants evaluated; Q1, first quartile; Q3, third quartile; RZV, recombinant zoster vaccine; VRR, vaccine response rate.

GMCs of anti-gE antibody at Month 0 preadditional vaccination were similar in all randomized groups: 10 149.5 mIU/mL in the 1-additional dose group, 11 548.5 mIU/mL in the revaccination group, and 10 232.0 mIU/mL in the control group (Figure 3*A*). Geometric mean concentrations peaked at 1 month after 1 vaccine dose, rising to 73 834.4 mIU/mL in the 1-additional dose group and 79 419.8 mIU/mL in the revaccination group (Figure 3*A*). In the control group, the GMC was stable at 9655.2 mIU/mL (Figure 3*A*). Geometric mean concentration values did not increase further at 1 month after the second vaccine dose in the revaccination group (64 603.0 mIU/mL) (Figure 3*A*). Geometric mean concentration levels declined at Year 1, with values of 24 663.6 and 26 167.5 mIU/mL, respectively, in the 1-additional dose and revaccination groups. Thereafter, GMCs remained at above preadditional vaccination Month 0 levels until the end of the study, with values of 12 868.4 and 13 534.0 mIU/mL, respectively, in the 1-additional dose and revaccination groups at Year 6 (Figure 3*A*). Geometric mean concentrations in the control group were 8825.4 mIU/mL at Year 1 and 6858.8 mIU/mL at Year 6 (Figure 3*A*).

Throughout the study, VRRs remained at above 84.0% in the 1-additional dose group and above 87.2% in the revaccination group, which were higher than in the control group (Figure 3*B*). At 1 month after 1 vaccine dose, there was a 58.2-fold MGI in the 1-additional dose group and a 78.4-fold MGI in the revaccination group (ratio of antibody concentration postvaccination vs prevaccination in ZOE-50/70; Figure 3*C*). At 1 month after the second vaccine dose in the revaccination group, MGI was 63.8-fold (Figure 3*C*). Compared with prevaccination levels in the ZOE-50/70 studies, MGI in the control group remained at approximately 9-fold at Months 1 and 3. Mean geometric increases remained higher in the 1-additional dose and revaccination groups than in the control group throughout the study (Figure 3*C*).

In the 1-additional dose group, the median frequency of CD4[2+] T-cells per million CD4 T-cells increased from 701.1 at Month 0 preadditional vaccination to 2632.4 at 1 month postvaccination (Figure 3*D*). In the revaccination group, the median frequency was 675.9 preadditional vaccination, 2736.7 at 1 month after the first vaccine dose, and 1953.5 at 1 month after the second vaccine dose (Figure 3*D*; Supplementary Table 1). At Years 1 and 6 of the study, the median frequencies were 1209.2 and 930.2, respectively, in the 1-additional dose group, 1019.6 and 770.4, respectively, in the revaccination group, and 548.2 and 460.8, respectively, in the control group.

The median frequency of gE-specific memory B-cells per million memory B-cells in the 1-additional dose group was 600.0 at Month 0 preadditional vaccination, 7586.5 at 1 month postvaccination, 1871.5 at Year 1, and 788.0 at Year 6 (Figure 3*E*; Supplementary Table 2). In the revaccination group, the median frequency was 1060.0 preadditional vaccination, 8693.5 at 1 month after the first vaccine dose, 6363.5 at 1 month after the second vaccine dose, 2108.0 at Year 1, and 848.0 at Year 6 (Figure 3*E*). The median frequency in the control group remained stable throughout the study, with minimal variation (707.0 at Month 0, 649.0 at Year 1, and 544.5 at Year 6).

### Reactogenicity and Safety

#### Postvaccination Solicited Adverse Events

In the 1-additional dose group, injection site pain was reported by 68.9% of participants, redness by 21.3%, and swelling by 11.5% (Table 2). Grade 3 pain, redness, and swelling were reported by 1.6%, 6.6%, and 1.6%, respectively. The median duration of pain was 3 days, redness 3 days and swelling 2 days. Fatigue, myalgia, shivering, and headache were the most common solicited systemic AEs, reported by 52.5%, 44.3%, 41.0%, and 37.7%, respectively, of participants in the 1-additional dose group (Table 2). The median duration of fatigue was 2 days, myalgia 2 days, shivering 1 day and headache 1 day. Shivering was the only grade 3 systemic AE reported (by 3.3% of participants).

**Table 2.** Adverse Events Experienced by Participants (TVC)

Solicited AEs Occurring In 7-d Postvaccination Period						
	1-Additional Dose Group N = 61		Revaccinated Group, Dose 1 N = 60		Revaccinated group, dose 2 N = 55	
	n	% (95% CI)	n	% (95% CI)	n	% (95% CI)
Injection site reaction						
Pain	42	68.9 (55.7, 80.1)	52	86.7 (75.4, 94.1)	37	67.3 (53.3, 79.3)
Grade 3^a^	1	1.6 (0.0, 8.8)	3	5.0 (1.0, 13.9)	2	3.6 (0.4, 12.5)
Redness	13	21.3 (11.9, 33.7)	20	33.3 (21.7, 46.7)	20	36.4 (23.8, 50.4)
Grade 3^a^	4	6.6 (1.8, 15.9)	1	1.7 (0.0, 8.9)	3	5.5 (1.1, 15.1)
Swelling	7	11.5 (4.7, 22.2)	12	20.0 (10.8, 32.3)	13	23.6 (13.2, 37.0)
Grade 3^a^	1	1.6 (0.0, 8.8)	0	0.0 (0.0, 6.0)	1	1.8 (0.0, 9.7)
Systemic reaction						
Fatigue	32	52.5 (39.3, 65.4)	24	40.0 (27.6, 53.5)	22	40.0 (27.0, 54.1)
Grade 3^a^	0	0.0 (0.0, 5.9)	2	3.3 (0.4, 11.5)	3	5.5 (1.1, 15.1)
Myalgia	27	44.3 (31.5, 57.6)	22	36.7 (24.6, 50.1)	12	21.8 (11.8, 35.0)
Grade 3^a^	0	0.0 (0.0, 5.9)	2	3.3 (0.4, 11.5)	1	1.8 (0.0, 9.7)
Shivering	25	41.0 (28.6, 54.3)	15	25.0 (14.7, 37.9)	7	12.7 (5.3, 24.5)
Grade 3^a^	2	3.3 (0.4, 11.3)	4	6.7 (1.8, 16.2)	0	0.0 (0.0, 6.5)
Headache	23	37.7 (25.6, 51.0)	17	28.3 (17.5, 41.4)	15	27.3 (16.1, 41.0)
Grade 3^a^	0	0.0 (0.0, 5.9)	2	3.3 (0.4, 11.5)	1	1.8 (0.0, 9.7)
Temperature ≥37.5°C	18	29.5 (18.5, 42.6)	13	21.7 (12.1, 34.2)	7	12.7 (5.3, 24.5)
>39.0°C	0	0.0 (0.0, 5.9)	0	0.0 (0.0, 6.0)	0	0.0 (0.0, 6.5)
GI symptoms	9	14.8 (7.0, 26.2)	9	15.0 (7.1, 26.6)	4	7.3 (2.0, 17.6)
Grade 3^a^	0	0.0 (0.0, 5.9)	2	3.3 (0.4, 11.5)	0	0.0 (0.0, 6.5)

Unsolicited AEs in 30-d Postvaccination Period^b^						
	1-Additional Dose Group N = 61		Revaccinated Group^c^ N = 60		Control group N = 119	
	n	% (95% CI)	n	% (95% CI)	n	% (95% CI)
Any	13	21.3 (11.9, 33.7)	15	25.0 (14.7, 37.9)	NA	NA
Grade 3^a^	0	0.0 (0.0, 5.9)	4	6.7 (1.8, 16.2)	NA	NA
Related	4	6.6 (1.8, 15.9)	4	6.7 (1.8, 16.2)	NA	NA
Medically attended	6	9.8 (3.7, 20.2)	7	11.7 (4.8, 22.6)	17	14.3 (8.5, 21.9)
SAEs	0	0.0 (0.0, 5.9)	2	3.3 (0.4, 11.5)	5	4.2 (1.4, 9.5)
pIMDs	0	NA	0	NA	0	NA

Abbreviations: AE, adverse event; CI, confidence interval; GI, gastrointestinal; N, number of participants in each group; n, number of participants with event; NA, not applicable; pIMD, potential immune-mediated disease; SAE, serious adverse event; TVC, total vaccinated cohort.

^a^Grade 3 redness and swelling were defined as an area >100 mm, all other grade 3 AEs were defined as preventing normal activity.

^b^30-d postvaccination period for the 1-additional dose and revaccination groups and within 30 d of the Month 0 and Month 2 visits for the control group.

^c^Data for both doses combined.

Following the first additional dose in the revaccination group, 86.7% of participants reported pain (grade 3, 5.0%), 33.3% reported redness (grade 3, 1.7%), and 20.0% reported swelling (no grade 3 swelling was reported); fatigue, myalgia, headache, and shivering were reported by 40.0% (grade 3, 3.3%), 36.7% (grade 3, 3.3%), 28.3% (grade 3, 3.3%), and 25.0% (grade 3, 6.7%) of participants, respectively (Table 2). Following the second additional vaccination in the revaccination group, pain, redness, and swelling were reported by 67.3% (grade 3, 3.6%), 36.4% (grade 3, 5.5%), and 23.6% (grade 3, 1.8%), respectively; fatigue, myalgia, headache, and shivering were reported by 40.0% (grade 3, 5.5%), 21.8% (grade 3, 1.8%), 27.3% (grade 3, 1.8%), and 12.7% (no grade 3 shivering was reported) (Table 2). The median duration of pain across both doses in the revaccination group was 2 days, redness 3 days and swelling 3 days. The median duration of fatigue was 2 days, myalgia 1 day, headache 2 days and shivering 1 day.

#### Unsolicited Adverse Events

In the 30-day postvaccination period, 21.3% of participants in the 1-additional dose group and 25.0% in the revaccination group reported any unsolicited AE; related AEs were reported in 6.6% and 6.7%, respectively (Table 2). During the same period, medically attended AEs were reported by 9.8% of the 1-additional dose group and 11.7% of the revaccination group (Table 2). Serious adverse events were reported by 3.3% of participants in the revaccination group, with none reported in the 1-additional dose group (Table 2). No pIMDs were reported.

Medically attended AEs were reported by 21.3% of participants in the 1-additional dose group and 20.0% of the revaccination group up to 6 months post last vaccination, and by 38.7% of participants in the control group up to 6 months after the Month 0 visit. Serious adverse events were reported in 4 participants (6.6%) in the 1-additional dose group and 4 participants (6.7%) in the revaccination group up to 12 months post last vaccination, and in 11 participants (9.2%) in the control group up to 12 months after the Month 0 visit. No pIMDs were reported during the same period. No SAEs related to RZV, study participation, or concurrent GSK medication or vaccine were reported throughout the entire study. Withdrawals due to an SAE or AE were reported in 3 participants in the 1-additional dose group (all SAEs), 1 participant in the revaccination group (nonserious AE), and 9 participants in the control group (all SAEs). There were no withdrawals resulting from HZ-associated complications. Two participants in the revaccination group did not receive the second vaccine dose due to a solicited AE following the first dose.

## DISCUSSION

The primary objective of ZOE-LTFU was to evaluate long-term VE in participants who received no additional RZV doses. The study showed that RZV offered durable protection against HZ and associated complications through 11 years postvaccination, with minimal waning of VE [9]. The high VE demonstrated throughout ZOE-LTFU means that the need for an additional dose(s) of RZV is yet to be established. Nevertheless, if and when the need is established, it is important to understand the impact of 1 or 2 additional doses of RZV on immunogenicity and safety.

Administration of additional vaccine doses is expected to elicit a strong and rapid anamnestic immune response, indicative of a functioning immune memory at the time of the additional dose. In the present analysis of ZOE-LTFU, 1 or 2 additional doses of RZV were administered approximately 5–6 years after primary vaccination in the ZOE-50/70 studies. The results show that a single additional dose of RZV elicited strong humoral and CMI anamnestic responses, with no changes to the known benefit/risk profile established in the primary studies.

In the present study, anti-gE antibody titers before the additional vaccination(s) were within the same range in all 3 randomized groups. At 1 month postvaccination, titers rose approximately 7-fold above preadditional vaccination levels at the start of ZOE-LTFU, to levels of 73 834.4 mIU/mL in the 1-additional dose group and 79 419.8 mIU/mL in the revaccination group. This is higher than the levels reached after primary vaccination in the ZOE-50/70 studies (approximately 50 000 mIU/mL) [11]. Antibody titers did not increase further after the second vaccine dose in the revaccination group. By Year 1, titers had declined to 24 663.6 mIU/mL in the 1-additional dose group and to 26 167.5 mIU/mL in the revaccination group. In the control group, antibody titers were similar at Year 1 to those at the corresponding time point in the much larger group of participants in ZOE-LTFU who received no additional vaccination and were evaluated for efficacy [9].

The T-cell response is believed to be more important than the humoral response for protection against HZ [12]. The median frequency of gE-specific CD4[2+] T-cells rose by approximately 4-fold at 1 month postvaccination compared with preadditional vaccination in ZOE-LTFU in both the 1-additional dose and revaccination groups, to 2632.4 and 2736.7. As with HI, this is higher than seen after primary vaccination (less than 2000) [11]. There was no further increase after the second additional dose. At Year 1, median frequencies declined to 1209.2 and 1019.6 in the 1-additional dose and revaccination groups, respectively.

The humoral and cell-mediated data up to Year 1 confirm data from a phase 2 study in which participants ≥60 years were vaccinated with 1 or 2 additional doses of RZV at 10 years after primary vaccination [13]. In that study, as in the present one, strong anamnestic responses were elicited by 1 additional dose of vaccine, with no further increase after a second additional dose [13]. Both antibody titers and CD4[2+] T-cell frequency followed a similar pattern to the present study ie, peaked at 1 month after an additional vaccine dose and declined after 1 year, but remained above preadditional vaccination levels [13].

The present study expands upon the phase 2 study by providing data on the durability of the anamnestic response to Year 6 after additional vaccination. At Year 6 of ZOE-LTFU, antibody titers and frequency of gE-specific CD4[2+] T-cells in the 1-additional dose and revaccination groups remained above preadditional vaccination levels at the start of ZOE-LTFU and above those in the control group who received no vaccination. Antibody titers and frequency of gE-specific CD4[2+] T-cells in the control group at Year 6 remained above baseline levels in the ZOE-50/70 studies before primary vaccination [11] and were similar at the corresponding time point to participants in ZOE-LTFU who received no additional vaccination and were evaluated for efficacy [9].

Memory B-cell evaluation was not performed in the phase 2 study [13], and the current data generated in ZOE-LTFU provide novel information. The gE-specific memory B-cell frequencies rose by approximately 8- to 12-fold at 1 month postvaccination versus prevaccination, without additional increase after the second additional dose. The sharp increase observed after a single additional dose supports the observation made with antibody levels and represents a hallmark of anamnestic response. Strong anamnestic responses can be induced even decades after the primary vaccination regimen by vaccines that offer long-term protection such as the hepatitis B virus vaccine [14], demonstrating the long-lived immune memory installed by the initial vaccination. The present study demonstrates that a single additional RZV dose induces robust cellular and humoral immunity comparable to those observed after primary vaccination, suggesting that RZV is also able to induce such a long-term immune memory. This likely plays an important role in the prolonged protection against HZ offered by RZV, as demonstrated in ZOE-LTFU [9].

The additional doses of RZV were well tolerated and demonstrated no changes in the known reactogenicity and safety profile established in the primary studies [15]. The benefit/risk profile was in line with that established in pivotal studies both in participants in the revaccination group who received 2 additional doses and those in the 1-additional dose group who received 1 additional dose. The most frequently reported solicited AEs were injection site pain, fatigue, and myalgia. There were few grade 3 AEs. No related deaths or SAEs, or related AEs or SAEs leading to withdrawal, were reported during the entire study.

Based on data from the ZOE-LTFU study showing that estimated VE of RZV is maintained at 82% at 11 years postvaccination [9], the need for an additional dose is not yet established. The potential need is being actively investigated, considering long-term protection (clinical efficacy and real-world effectiveness), evaluation of breakthrough cases, persistence of the immune response, and modeling of the immune response.

The present analysis had some limitations. It was descriptive in nature and was designed only to observe the immune response to additional doses of RZV. It did not attempt to evaluate how the immune response to additional doses impacts VE. The analysis was conducted on a small subset of participants from the entire study and included only those from 3 preselected countries. Almost all participants were of White European origin which could limit its generalizability to wider populations.

In conclusion, although the need for additional vaccination with RZV is not yet established, the study demonstrated that a single additional dose of RZV elicited strong humoral and CMI anamnestic responses, with a reactogenicity and safety profile in line with that established in pivotal studies. Importantly, memory B-cell data indicate that primary vaccination with RZV induces a durable memory response, which might underlie minimal waning of VE over time.

## Supplementary Material

ofag282_Supplementary_Data

## Supplementary Data

Supplementary materials are available at *Open Forum Infectious Diseases* online. Consisting of data provided by the authors to benefit the reader, the posted materials are not copyedited and are the sole responsibility of the authors, so questions or comments should be addressed to the corresponding author.

## References

[1] Mueller NH, Gilden DH, Cohrs RJ, Mahalingam R, Nagel MA. Varicella zoster virus infection: clinical features, molecular pathogenesis of disease, and latency. Neurol Clin 2008; 26:675–97. viii. 10.1016/j.ncl.2008.03.011 PMC2754837 18657721

[2] Yawn BP, Saddier P, Wollan PC, et al A population-based study of the incidence and complication rates of herpes zoster before zoster vaccine introduction. Mayo Clin Proc 2007; 82:1341–9. 10.4065/82.11.1341 17976353

[3] Centers for Disease Control and Prevention . Clinical features of shingles (herpes zoster). 2024. Available at: https://www.cdc.gov/shingles/hcp/clinical-signs/index.html (accessed 16 November 2024).

[4] Johnson RW, Alvarez-Pasquin M-J, Bijl M, et al Herpes zoster epidemiology, management, and disease and economic burden in Europe: a multidisciplinary perspective. Ther Adv Vaccines 2015; 3:109–20. 10.1177/2051013615599151 PMC4591524 26478818

[5] United Nations . Department of Economic and Social Affairs. World social report 2023: leaving no one behind in an ageing world. 2023. Available at: https://desapublications.un.org/publications/world-social-report-2023-leaving-no-one-behind-ageing-world (accessed 23 November 2023).

[6] Chlibek R, Bayas JM, Collins H, et al Safety and immunogenicity of an AS01-adjuvanted varicella-zoster virus subunit candidate vaccine against herpes zoster in adults ≥50 years of age. J Infect Dis 2013; 208:1953–61. 10.1093/infdis/jit365 23904292

[7] Cunningham AL, Lal H, Kovac M, et al Efficacy of the herpes zoster subunit vaccine in adults 70 years of age or older. N Engl J Med 2016; 375:1019–32. 10.1056/NEJMoa1603800 27626517

[8] Lal H, Cunningham AL, Godeaux O, et al Efficacy of an adjuvanted herpes zoster subunit vaccine in older adults. N Engl J Med 2015; 372:2087–96. 10.1056/NEJMoa1501184 25916341

[9] Strezova A, Díez Domingo J, Cunningham AL, et al Final analysis of the ZOE-LTFU trial to 11 years post-vaccination: efficacy of the adjuvanted recombinant zoster vaccine against herpes zoster and related complications. EClinicalMedicine 2025; 83:103241. 10.1016/j.eclinm.2025.103241 PMC12235393 40630610

[10] Didierlaurent AM, Laupèze B, Di Pasquale A, Hergli N, Collignon C, Garçon N. Adjuvant system AS01: helping to overcome the challenges of modern vaccines. Expert Rev Vaccines 2017; 16:55–63. 10.1080/14760584.2016.1213632 27448771

[11] Cunningham AL, Heineman TC, Lal H, et al Immune responses to a recombinant glycoprotein E herpes zoster vaccine in adults aged 50 years or older. J Infect Dis 2018; 217:1750–60. 10.1093/infdis/jiy095 PMC5946839 29529222

[12] Arvin A . Aging, immunity, and the varicella-zoster virus. N Engl J Med 2005; 352:2266–7. 10.1056/NEJMp058091 15930416

[13] Hastie A, Catteau G, Enemuo A, et al Immunogenicity of the adjuvanted recombinant zoster vaccine: persistence and anamnestic response to additional doses administered 10 years after primary vaccination. J Infect Dis 2021; 224:2025–34. 10.1093/infdis/jiaa300 PMC8672743 32502272

[14] Van Damme P, Dionne M, Leroux-Roels G, et al Persistence of HBsAg-specific antibodies and immune memory two to three decades after hepatitis B vaccination in adults. J Viral Hepat 2019; 26:1066–75. 10.1111/jvh.13125 PMC6852111 31087382

[15] López-Fauqued M, Campora L, Delannois F, et al Safety profile of the adjuvanted recombinant zoster vaccine: pooled analysis of two large randomised phase 3 trials. Vaccine 2019; 37:2482–93. 10.1016/j.vaccine.2019.03.043 30935742

