# Correction to: Safety and Immunogenicity of 1 or 2 Additional Doses of the Adjuvanted Recombinant Zoster Vaccine Administered 5–6 Years After Primary Vaccination in Adults ≥50 Years

**DOI:** 10.1093/ofid/ofag695

**Published:** 2026-10-31

This is a correction to: Rafael Leon, Javier Diez-Domingo, Charles Andrews, Clovis Arns da Cunha, Eun-Ju Choo, David Shu Cheong Hui, Anthony L Cunningham, Takashi Eto, Giancarlo Icardi, Shelly A McNeil, Airi Põder, Pavel Kosina, Lars Rombo, Tino F Schwarz, Juan Carlos Tinoco, Chong-Jen Yu, Jing Wang, Jyoti Soni, Manyee Tsang, Ana Strezova, Bruno Salaun, Agnes Mwakingwe-Omari, on behalf of the Zoster-049 Study Group, Safety and Immunogenicity of 1 or 2 Additional Doses of the Adjuvanted Recombinant Zoster Vaccine Administered 5–6 Years After Primary Vaccination in Adults ≥50 Years, *Open Forum Infectious Diseases*, Volume 13, Issue 6, June 2026, ofag282, https://doi.org/10.1093/ofid/ofag282

In the originally published version of this manuscript, the list of study group members was omitted from the ‘Notes’ section and erroneously included in the supplementary material.

The study group members are now listed under the ‘Notes’ heading.

