# Supplementary material for: Safety and Immunogenicity of 1 or 2 Additional Doses of the Adjuvanted Recombinant Zoster Vaccine Administered 5–6 Years After Primary Vaccination in Adults ≥50 Years
Source: Open Forum Infect Dis. 2026 May 4;13(6):ofag282. doi: 10.1093/ofid/ofag282 (PMC13232749; doi:10.1093/ofid/ofag282)
Supplement: ofag282_Supplementary_Data [file ofag282_supplementary_data.zip › study group zoster-049_list.docx]

## **Study group**

**Investigators:** Abul Kashem Munir, Agnes Csuth, Agnes Himpel-Boenninghoff, Aino Forsten, Airi Poder, Ake Olsson, Alain Baty, Alain Boye, Alen Jambrecina, Alex Rodríguez Badia, Alexander Thompson, Andrea Gori, Anitta Ahonen, Anna Vilella Morato, Anthony Cunningham, Antje Dahmen, Axel Schaefer, Azhar Toma, Barry Lubin, Beate Moeckesch, Beatrice Gerlach, Benita Ukkonen, Benjamin Lasko, Benoit Daguzan, Bernhard Schmitt, Bo Liu, Brian Webster, Bruce Rankin, Calvin Powell, Carlos Brotons Cuixart, Carol Pretswell, Catherine Vaillancourt, Cecil Farrington, Charles Andrews, Chester Fisher, Chigomezgo Munthali, Chiu-Shong Liu, Chong-Jen Yu, Christian Duroy, Christian Schubert, Christiane Klein, Christine Cerna, Christine Grigat, Christophe Genies, Christopher Lucasti, Cláudia Murta de Oliveira, Claus Keller, Clovis Cunha, Concepcion Nunez Lopez, Covadonga Caso, Cristiano Zerbini, Dae Won Park, Damien Mcnally, Dan Curiac, David Francyk, David García Vidal, David Shu Cheong Hui, Denis Taminau, Domenico Montu, Dominique Saillard, Donald Quinn, Duane Wombolt, Edmund Kwok Yiu Sha, Elina Sirnela-Rif, Elisabeth Barberan, Eric St-Amour, Eriko Kinugasa, Ernie Riffer, Essam Abdulhakim, Eugene Athan, Eun-Ju Choo, Eva Ackefelt Frick, Felix Proepper, Ferdinandus de Looze, Francesco Schioppa, François Brault, Frederick Martin, Gabriele Illies, Georg Plassmann, George Freeman, George Raad, Gerald Shockey, Giancarlo Icardi, Giuseppe Fiore, Guglielmo Migliorino, Guy Tellier, Hanna Karhusaari, Hans-Joachim Koenig, Hee Jin Cheong, Hiroaki Ogata, Hirohiko Sueki, Holly Dushkin, Hsiao-Ting Chang, Huey-Shinn Cheng, Hyo Youl Kim, Ignacio Bardón Fernández-Pacheco, Ilkka Seppa, Irina Zahharova, Iris Gorfinkel, Isaac Marcadis, Isabelle Schenkenberger, Jacob Lee, Jan Dutz, Janice Patrick, Javier Diez-Domingo, Jean Beytout, Jean-Sebastien Gauthier, Jeannette Janzen, Jeffrey Zacher, Jérôme Nuel, Jessica Claveau, Jin-Soo Lee, Joachim Minnich, Joachim Sauter, Joakim Aronsson, Joan Rothenberg, Johan Sanmartin Berglund, John Earl, John Ervin, Jonathan Staub, Jonathan Wilson, José Luiz Neto, José Ramón Méndez Rivas, Jose-Fernando Barba-Gómez, Josef Grosskopf, Juan Carlos Tinoco, Juergen Berger-Roscher, Juergen Schmidt, Juergen Stockhausen, Juergen Wachter, Jukka Markkula, Junya Irimajiri, Jurij Eremenko, Kae Kobayashi, Karl Wilhelm, Karlis Pauksens, Katarina Berndtsson Blom, Ken Heaton, Kenjiro Nakamura, Kyong Ran Peck, Lars Rombo, Lauri Peltonen, Laurie Breger, Lluis Martínez Via, Loïc Boucher, Luciano Goldani, M Luisa Rodrguez de la Pinta, Mahadev Ramjee, Maija Rossi, Manuel Terns Riera, Marc Dionne, Margaret Rhee, Maria Giuseppina Desole, Maria Hemming-Harlo, Maria Maestre Naranjo, Marita Paassilta, Marjaana Sipila, Mark Turner, Marshall Freedman, Marta Aldea Novo, Martin Lundvall, Martin Van Cleeff, Mary Beth Manning, Matthew Finneran, Maximilian Kropp, Megumi Inoue, Meral Esen, Merce Perez Vera, Michael Adams, Michael Mueller, Michael Redmond, Miia Virta, Monika Hamann, Murdo Ferguson, Nell Wyatt, Nicolas Galerne, Nicole Toursarkissian, Niklas Bengtsson, Noah Vale, Olli Henriksson, Otso Arponen, Outi Laajalahti, Pascal Hanrion, Patrice Nault, Patrick Robert, Paul Hartley, Paul Ivan, Paula Gyllemark, Pauliina Paavola, Pavel Kosina, Pavel Naplava, Pekka Koskinen, Pembe Ozunlu, Peter Eizenberg, Peter Gal, Peter Levins, Petr Dite, Philippe Remaud, Piero Barbanti, Pierre André Ferrand, Pierre Lachance, Pierre-Alain Houle, Pyrene Martinez Piera, Ralf Freese, Rie Kuroki, Robert Lipetz, Robert Rosen, Roman Chlibek, Samir Purnell-Mullick, Satu Kokko, Scott Polster, Shari Rozen, Shelly McNeil, Shin Suzuki, Shinn-Jang Hwang, Silvia Narejos Perez, Spyridon Miyakis, Srikanth Malempati, Stephan Morscher, Stephanie Powell, Steve Mueller, Steven Geller, Suganthi Luci Magimaiseelan, Susan Datta, Susanna Koski, Susannah Eyre, Susanne Hoeltz-Roehrig, Suvi-Tuuli Simojoki, Sylvia Shoffner, Takashi Eto, Tamara Eckermann, Tark Kim, Terry Poling, Tetsuhiko Nagao, Thomas Horacek, Thomas Jung, Thomas Weinke, Tiina Haapaniemi, Tiina Karppa, Tiina Korhonen, Tino Schwarz, Tommaso Staniscia, Trevor Gooding, Uwe Kleinecke, Wayne Ghesquiere, Wilfred Yeo, William Ellison, Wilson Jacob, Xavier Farres Fabre, Yieng Huong, Young Goo Song, Yuji Naritomi

**GSK:** Agnes Mwakingwe-Omari, Alemnew F Dagnew, Amy Tan, Ana Strezova, Anne Schuind, Andrew Hastie, Bruno Salaun, Céline Boutry, Emeline de Viron, Emmanuel Di Paolo, Hao Wang, Huizi Zhang, Joon Hyung Kim, Kamal Al Shawafi, Lidia Oostvogels, Mamadou Drame, Martine Douha, Mélanie Gilbert, Meng Shi, Mohamed Amakrane, Mohd Tariq, Nurhan Albayrak, Olivier Godeaux, Paola Pirrotta, Toufik Zahaf
