# Supplementary material for: Safety and Immunogenicity of 1 or 2 Additional Doses of the Adjuvanted Recombinant Zoster Vaccine Administered 5–6 Years After Primary Vaccination in Adults ≥50 Years
Source: Open Forum Infect Dis. 2026 May 4;13(6):ofag282. doi: 10.1093/ofid/ofag282 (PMC13232749; doi:10.1093/ofid/ofag282)
Supplement: ofag282_Supplementary_Data [file ofag282_supplementary_data.zip › Supplementary Appendix_REVISED_clean.docx]

**Supplementary Appendix**

**Safety and immunogenicity of one or two additional doses of the adjuvanted recombinant zoster vaccine administered 5–6 years after primary vaccination in adults ≥50 years**

Leon R, et al, on behalf of the Zoster-049 Study Group

**Supplementary methods**

## Exclusion criteria for 1-additional dose, revaccination, and control groups

Participants who experienced a serious adverse event from the first vaccination in the previous ZOE-50/70 studies to enrollment in the ZOE-LTFU study that was considered related to the study vaccine by either the investigator or the sponsor.

Participants with a new onset of a potential immune-mediated disease (pIMD) or exacerbation of a pIMD from the first vaccination in the previous ZOE-50/70 studies to enrollment in the ZOE-LTFU study.

Use of any investigational or non-registered product (pharmaceutical product or device) within 30 days preceding the first dose of the study vaccine or planned use during the study period.

Administration or planned administration of any other immunizations within 30 days before the first study vaccination or scheduled within 30 days after study vaccination. However, licensed non-replicating vaccines (i.e., inactivated and subunit vaccines, including inactivated and subunit influenza vaccines for seasonal or pandemic influenza, with or without adjuvant) were permitted to be administered up to 8 days prior to each dose and/or at least 14 days after any dose of the study vaccine.

History of allergic disease or reactions likely to be exacerbated by any component of the vaccine or by other material or equipment related to study participation (such as materials that might contain latex, e.g., gloves, syringes, etc.). The vaccine and vials in this study did not contain latex.

Pregnant or lactating female.

Females of childbearing potential planning to become pregnant or planning to discontinue contraceptive precautions.

Previous episode or history of herpes zoster.

Participants who were part of the immunogenicity or cell-mediated immunity subsets of ZOE-50/70.

Participants who were not part of the according-to-protocol cohort for the analysis of vaccine efficacy in ZOE-50/70.

## Assay methodology for memory B-cells

Varicella zoster virus (VZV) glycoprotein E (gE) antigen was fluorescently labeled with either DyLight 550 or DyLight 650 dyes according to the manufacturer’s instructions (ThermoFisher). Frozen peripheral blood mononuclear cells were thawed, and 4.10^6^ cells were stained with viability dye, phenotypic surface markers (CD14, IgD, CD19, CD20, CD38) and fluorescently labeled VZV gE probes. Flow cytometry data were analyzed using FlowJo software (Becton Dickinson). Memory B-cells were defined as live CD14^-^/IgD^-^/CD19^+^/CD20^+^/CD38^low^ cells. VZV gE-specific memory B-cells are memory B-cells identified by double staining with the two fluorescent probes. The gating strategy and assay quality control (specificity) are shown in Supplementary Figure S1, demonstrating that increased VZV gE-specific memory B-cell frequencies are only detected with the VZV gE probe (and not with an irrelevant antigen probe) after recombinant zoster vaccine immunization.

Supplementary Figure S1. Assay methodology for memory B-cells: (A) Gating strategy applied to detect VZV gE-specific memory B-cells; (B) VZV gE-specific memory B-cell assay specificity

**A**


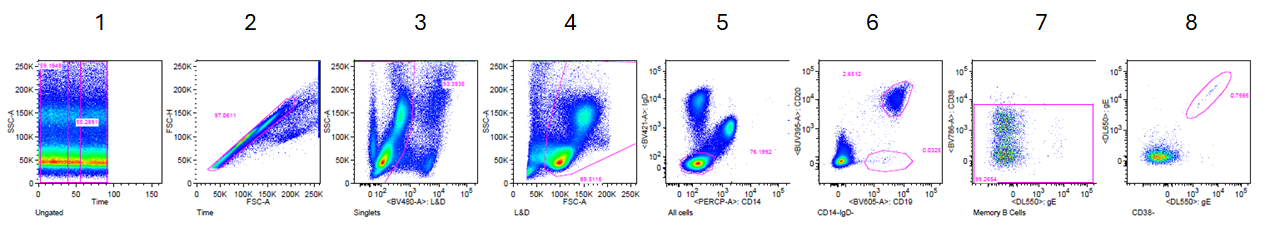


From cells acquired at stable flow rate (1), doublets were excluded (2) and only live cells were analyzed (3). After cell debris exclusion (4), IgD^-^ CD14^-^ were selected (5), and CD19^+^ CD20^+^ B-cells (6) were then gated to exclude CD38^high^ cells (7). VZV gE specific memory B-cells were then detected using VZV gE antigen baits coupled to either DyLight 550 (DL550) or DyLight 650 (DL650), and only double positive memory B-cells were considered to be VZV gE specific (8)

**B**


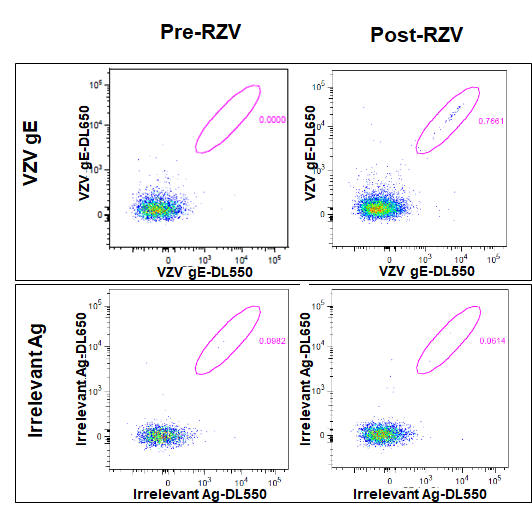


To confirm assay specificity, PBMC collected from RZV recipients before and after immunization were analyzed with either the VZV gE protein or an irrelevant protein antigen similarly coupled to DyLight fluorescent dyes as a probe. A representative example is shown (gating on live CD14^-^/IgD^-^/CD19^+^/CD20^+^/CD38^low^ cells).

Ag, antigen; DL, Dylight; gE, glycoprotein E; L&D, live dead viability marker; PBMC, peripheral blood mononuclear cells; RZV, recombinant zoster vaccine; VZV, varicella zoster virus

Supplementary Table S1. Frequency of gE-specific CD4[2+] T-cells per million CD4 T-cells following additional RZV doses (adapted ATP cohort for immunogenicity)

| **Timepoint** | **N** | **Minimum** | **Q1** | **Median** | **Q3** | **Maximum** |
| --- | --- | --- | --- | --- | --- | --- |
| **1-additional dose group** | | | | | | |
| M0 | 55 | 33.1 | 396.0 | 701.1 | 1118.2 | 4386.6 |
| M1 | 52 | 1.0 | 1614.8 | 2632.4 | 6030.9 | 15,519.6 |
| Year 1 | 53 | 1.0 | 583.9 | 1209.2 | 2311.3 | 14,221.2 |
| Year 2 | 58 | 1.0 | 506.8 | 988.8 | 2187.1 | 12,393.1 |
| Year 3 | 51 | 1.0 | 600.5 | 1351.5 | 2918.4 | 16,791.0 |
| Year 4 | 45 | 1.0 | 680.9 | 1343.8 | 2763.5 | 15,321.6 |
| Year 5 | 48 | 1.0 | 490.2 | 873.6 | 2045.0 | 15,960.9 |
| Year 6 | 48 | 1.0 | 418.1 | 930.2 | 1997.2 | 17,073.7 |
| **Revaccination group** | | | | | | |
| M0 | 54 | 10.0 | 273.0 | 675.9 | 1216.7 | 4443.2 |
| M1 | 46 | 100.5 | 1597.4 | 2736.7 | 3854.7 | 7071.8 |
| M3 | 51 | 383.1 | 1117.3 | 1953.5 | 2635.7 | 28,900.1 |
| Year 1 | 50 | 1.0 | 644.7 | 1019.6 | 1597.8 | 3950.5 |
| Year 2 | 46 | 1.0 | 595.7 | 990.7 | 1603.0 | 3798.6 |
| Year 3 | 45 | 1.0 | 715.8 | 1051.6 | 1557.8 | 4310.2 |
| Year 4 | 43 | 1.0 | 618.0 | 942.7 | 1603.1 | 2515.8 |
| Year 5 | 47 | 1.0 | 363.6 | 749.4 | 1305.3 | 2254.1 |
| Year 6 | 41 | 1.0 | 352.1 | 770.4 | 1142.4 | 2681.1 |
| **Control group** | | | | | | |
| M0 | 109 | 1.0 | 335.9 | 586.3 | 995.5 | 4384.7 |
| M1 | 104 | 1.0 | 288.8 | 578.9 | 876.5 | 2772.7 |
| M3 | 107 | 1.0 | 278.5 | 624.9 | 916.6 | 3723.8 |
| Year 1 | 97 | 1.0 | 232.4 | 548.2 | 817.9 | 3268.4 |
| Year 2 | 109 | 1.0 | 218.4 | 519.6 | 945.1 | 3351.3 |
| Year 3 | 95 | 1.0 | 222.4 | 566.3 | 1019.0 | 5797.4 |
| Year 4 | 83 | 1.0 | 313.1 | 638.2 | 1118.4 | 3880.4 |
| Year 5 | 93 | 1.0 | 221.6 | 505.7 | 927.9 | 3387.4 |
| Year 6 | 82 | 1.0 | 217.4 | 460.8 | 1071.2 | 3262.4 |

ATP, according to protocol; M0, month 0 (before additional vaccination in the 1-additional dose and revaccination groups); M1, month 1 (1 month after the first additional vaccine dose in the 1-additional dose and revaccination groups); M3, month 3 (1 month after the second additional vaccine dose in the revaccination group); N, number of participants evaluated; Q1, first quartile; Q3, third quartile; RZV, recombinant zoster vaccine

Supplementary Table S2. Frequency of gE-specific memory B-cells per million memory B-cells following additional RZV doses (adapted ATP cohort for immunogenicity)

| **Timepoint** | **N** | **Minimum** | **Q1** | **Median** | **Q3** | **Maximum** |
| --- | --- | --- | --- | --- | --- | --- |
| **1-additional dose group** | | | | | | |
| M0 | 50 | 0.0 | 318.0 | 600.0 | 1203.0 | 3340.0 |
| M1 | 52 | 771.0 | 2773.5 | 7586.5 | 11612.0 | 22,234.0 |
| Year 1 | 46 | 0.0 | 707.0 | 1871.5 | 3331.0 | 9676.0 |
| Year 2 | 50 | 211.0 | 915.0 | 1524.5 | 2139.0 | 7607.0 |
| Year 3 | 48 | 0.0 | 499.5 | 958.0 | 1736.5 | 5863.0 |
| Year 4 | 39 | 0.0 | 349.0 | 723.0 | 1325.0 | 4092.0 |
| Year 5 | 43 | 0.0 | 337.0 | 770.0 | 1202.0 | 4446.0 |
| Year 6 | 44 | 0.0 | 354.0 | 788.0 | 1285.5 | 4180.0 |
| **Revaccination group** | | | | | | |
| M0 | 49 | 0.0 | 509.0 | 1060.0 | 1527.0 | 6436.0 |
| M1 | 46 | 1066.0 | 3636.0 | 8693.5 | 14098.0 | 45,124.0 |
| M3 | 46 | 832.0 | 3648.0 | 6363.5 | 8922.0 | 32,827.0 |
| Year 1 | 45 | 0.0 | 1073.0 | 2108.0 | 4113.0 | 18,124.0 |
| Year 2 | 47 | 0.0 | 678.0 | 1574.0 | 2494.0 | 11,286.0 |
| Year 3 | 43 | 0.0 | 698.0 | 1134.0 | 1919.0 | 9724.0 |
| Year 4 | 40 | 0.0 | 543.5 | 965.0 | 2105.5 | 8691.0 |
| Year 5 | 41 | 0.0 | 539.0 | 908.0 | 1556.0 | 4514.0 |
| Year 6 | 38 | 0.0 | 506.0 | 848.0 | 1514.0 | 4270.0 |
| **Control group** | | | | | | |
| M0 | 96 | 0.0 | 438.0 | 707.0 | 1163.5 | 4378.0 |
| M1 | 94 | 0.0 | 436.0 | 774.0 | 1211.0 | 3865.0 |
| M3 | 98 | 0.0 | 327.0 | 775.0 | 1316.0 | 3899.0 |
| Year 1 | 84 | 0.0 | 321.0 | 649.0 | 1111.0 | 2656.0 |
| Year 2 | 101 | 0.0 | 295.0 | 630.0 | 962.0 | 3825.0 |
| Year 3 | 99 | 0.0 | 238.0 | 444.0 | 896.0 | 3602.0 |
| Year 4 | 83 | 0.0 | 316.0 | 531.0 | 808.0 | 3102.0 |
| Year 5 | 89 | 0.0 | 322.0 | 517.0 | 792.0 | 2427.0 |
| Year 6 | 78 | 0.0 | 274.0 | 544.5 | 959.0 | 5207.0 |

ATP, according to protocol; M0, month 0 (before additional vaccination in the 1-additional dose and revaccination groups); M1, month 1 (1 month after the first additional vaccine dose in the 1-additional dose and revaccination groups); M3, month 3 (1 month after the second additional vaccine dose in the revaccination group); N, number of participants evaluated; Q1, first quartile; Q3, third quartile; RZV, recombinant zoster vaccine
